# Wildlife and Newspaper Reporting in Iran: A Data Analysis Approach

**DOI:** 10.3390/ani11061487

**Published:** 2021-05-21

**Authors:** Farshad Amiraslani, Deirdre Dragovich

**Affiliations:** 1 School of Geography & Environmental Sciences, Faculty of Life & Health Sciences, Ulster University, Coleraine BT52 1SA, UK; 2 School of Geosciences, University of Sydney, Sydney, NSW 2006, Australia; deirdre.dragovich@sydney.edu.au

**Keywords:** wildlife, newspapers, Iran, SQL

## Abstract

**Simple Summary:**

Three major Iranian daily newspapers were analysed for news items relating to wildlife, covering a 7-year study period. Wildlife items were characterised by public awareness (51%), columnist contributions (46%), and local spatiality (43%). Most items (82%) were allocated space of less than half a page. Results highlighted the minimal number and small space devoted to wildlife news items in Iranian newspapers.

**Abstract:**

Human response to wildlife management is widespread, encompassing both human–wildlife conflicts and wildlife conservation, in different places and at different times. As people become increasingly aware of the importance of wildlife to biological and environmental sustainability, newspapers can be important sources of information, especially in developing countries, such as Iran. Three major Iranian daily newspapers were analysed for news items related to wildlife. Over the 7-year study period, 434 articles presented environmental news, of which 61 items referred to wildlife. Each wildlife item was recorded in terms of message, contributor, spatiality, and allocated space. Structure query language (SQL) was used to analyse relationships between the 915 fields/entries. Wildlife items were characterised by public awareness (51%), columnist contributions (46%), and local spatiality (43%). Most items (82%) were allocated space of less than half a page. Of the categorised topics, those of combined endangered land (30%) and marine (5%) species exceeded items on global conservation (24%). Results highlighted the minimal number and small space devoted to wildlife news items and their concentrations (67%) in one of the three sampled newspapers. Although nature has historically been important in Iranian culture, current attitudes to wildlife, as reflected in newspaper coverage, do not seem to mirror these traditional perspectives. Given the widespread distribution of newspapers and their roles (i.e., as sources of information and opinion influencers), global wildlife conservation issues would benefit from much greater coverage in the daily press.

## 1. Introduction

Contemporary urban populations encounter (and enjoy) wildlife—in one way or another—on a daily basis. For example, globally, wildlife are typically featured on aircrafts: the Kangaroo for Australian Qantas or the Oryx for Qatar Airways resonate pride in their countries’ native animal species. A visual content analysis of 637 global airlines indicated that 23% of aeroplanes’ tailfins represented an animal or a bird [1]. Iran Air aircrafts carry a blue-coloured mythical bird on their tailfins, a reminder of a rich heritage of Persian literature and history.

Urban areas have become locations for nesting, flying, breeding, and feeding wildlife, although animals may be distracted by night lights or noises, or be injured, poisoned, or killed because of human–animal interactions (for a Special Issue on behavioural and ecological consequences of urban life for birds, refer to [2]). In the context of global wildlife utilisation—their skin, feathers, ivories, horns, or bodies echo illegality of their presence in luxury clothes, footwear, medicines, artefacts, and foods (e.g., [3,4]). Moreover, the anthropogenic footprint jeopardises natural terrestrial and marine habitats. Despite the worldwide wildlife–human relationships, wildlife news items are not captured well by media.

Newspapers influence their readers through content, presentation, and positioning of individual news items and may transmit exaggerated, biased, or misleading news items that may threaten wildlife and wildlife conservation. As newspapers require a readership, editors can project their own perceptions of items or interpretations of public interests, while simultaneously influencing readers. In the absence of short-term prompting by reader activism or withdrawal of purchasing support, the newspaper–reader interaction effectively proceeds under the guidance of newspaper staff. Assessment of media, including newspapers, is an approach that researchers can employ to convert qualitative public information into quantitative data.

In animal conservation science, qualitative data can present valuable research findings, mainly when knowledge of the local language(s) and concepts exist [5]. In regards to the wildlife–human context, we seek to address this paucity in the global research domain, while finding answers to specific questions relevant to reporting of wildlife in Iranian newspapers. In addition to the uniqueness of this research, in terms of country coverage and topic, our paper employs a digital database management system, Structured Query Language (SQL), for data analysis. SQL commands allow the exploration of hidden relationships among the wording of news items published by print-based newspapers.

The questions posed in analysing Iranian newspapers were:

- Are local experts involved in writing technical articles or are these articles just being translated from international sources? (Contributor).
- What was the main message of an article in disseminating its wildlife news? (Message).
- What is the spatial focus of wildlife news in the sampled newspapers? (Spatiality).
- How much space is allocated for wildlife news in a sample of an Iranian newspaper? (Allocated space).

### 1.1. Wildlife and the Media

‘Environmental’ news items were among the news of least interest to readers, in general, in a study using computational approaches to extract data from large datasets (2.5 million articles from 498 different English language news outlets) [6]. However, the role of media in representing the climate change issue from various perspectives has attracted a growing number of papers globally (e.g., [7,8,9,10]). Comparatively, wildlife conservation, as a matter of environmental concern, has attracted minor attention among media analysts, even though it was equally challenging due to its associated unpredictable socio-economic outcomes. Using Google Trends to measure interest in conservation topics in online news (2004–2013), research showed a robust downward trend in public interest regarding endangered species [11]. While wild animals generally avoid direct encounters with humans, there are reports of human injuries and deaths due to depredations worldwide. Local people sometimes perceive wildlife and/or wildlife conservation plans as threats to their health, tranquillity, recreation, income, livestock, crops, lands, properties, buildings, and other assets; e.g., [12,13,14,15,16,17,18]. Wildlife conservation can sometimes become a disputed issue at a regional or international level, for example, whale hunting caused friction between Australia and Japan [19]. On a broader scale, the recent era coincided with an increase in human confrontation with wildlife, described as the ‘wildland–urban interface’ [20], caused by population growth, accelerated urbanization, intensified agriculture, deforestation, and habitat fragmentation, especially in developing countries (e.g., [21]). On a large scale, such issues have created ground for environmental crimes, including wildlife trafficking, which undermines global stability and peace [4].

Newspapers or other mass media are essential sources that reflect public attitudes and are powerful actors in shaping wildlife policies [22], although wildlife news and stories are rarely seen in the headlines [23]. The positive or negative views and ideas about wildlife issues will remain unclear to the public unless media outlets become involved or a local inquiry is launched, such as the urban population’s views on wildlife in Japan [24]. Even in developed countries, there are mixed reactions to whether legal hunting, lethal wildlife management, and gaming tourism are relevant and appropriate (e.g., for the USA: [25]). As such, the media can serve as crucial outlets for wildlife and biodiversity conservation [26,27]. According to the Eurobarometer survey conducted in 2007, “the most typical ways that Europeans learn about biodiversity issues are watching news and documentaries on TV, searching the Internet, and reading newspapers and magazines” ([28], p.8). The role of movies, featuring particular wildlife species and their impacts on the wildlife trade, has also been assessed, as reported in a research study observing links between the Harry Potter movies and the UK trade in owls [29].

The use of social media in wildlife conservation is attracting researchers in both natural and social sciences. In particular, social media’s future in addressing the grand challenges of biodiversity conservation seems promising [26]. This may be partly attributed to the growing number of social media users and the broad range of their interests. Users have a diverse spectrum of knowledge and background and their posts, comments, and photos can be obtained quickly and interactively shared at any time. People increasingly rely on media, which broadcast news in a frequent (temporal), broad (spatial), large (quantity), and fast (velocity) way. For instance, there were 99,000 reports per day when a trophy hunter killed a male lion in Zimbabwe [30]. Social media has also facilitated the growing trend of fashion-based presentation and normalization of destructive behaviours towards wildlife conservation, including the captivity of exotic animals by celebrities in the Middle East [31], and Facebook posts on shark coverage in Australia [32]. A limited number of studies have investigated the role of media in representing or misrepresenting news on various forms of wildlife and its conservation over the last three decades, including zebra mussels [33], cougars [22], panthers [18], spotted owls [34], and foxes [35].

### 1.2. Iran’s Perspective

Overall, the preceding literature review reveals a shortage of analytical assessments of global wildlife news, in particular, in western Asia, including Iran, despite the broader problem being acknowledged. Almost five decades ago, a report published by IUCN depicted a grim hunting picture for the Middle East region as a whole: “…it is rather surprising that so many wildlife species have managed to survive the onslaught of man without a serious contraction of their original range or a diminution of their numbers to the point that they are threatened by extinction” [36]. As one of the countries in this region, hunting for wildlife was documented for Iran. The only existing general article portraying wildlife in Iran assessed leopard mortality [37] and was based on unpublished data from organizations and Iranian websites.

Iran enjoys diverse climatic, geographical, and geological zones, which have created unique opportunities for wildlife, national parks, and human-made/natural forests and reserves across humid and dryland areas [38,39]. According to the Iran Department of Environment [40], the country encompasses 1131 animal species, including mammals, birds, reptiles, and amphibians across land and marine zones, of which some are endemic to Iran. The country was perhaps the first in the Middle East to include the word “hunting” in national legislation, as early as 1920 [41]. Legal hunting regulations were enacted in 1956 [41], but the lack of resources for field guards and the country’s vastness have deterred proper monitoring of legal and illegal hunting systems.

Throughout history, the glory of cultural, literary, and religious belongings, and the enthusiasm of Iranians for nature’s elements (such as water and plants) have resonated in both scientific [42] and historical scholarly manuscripts (e.g., for historical rain making ceremonies, songs, and rituals, refer to [43]). From a scientific perspective, Qanats or underground water canals, kilometres in length, were designed to preserve precious water resources throughout the year in dryland areas in Iran 3000 years ago [44]. Modern engineering and computation techniques have confirmed the possession of detailed and accurate skills among the earlier Iranians to construct these water canals [45]. Religious admiration, and keeping the water clean and safe, was also recorded: polluting water, in particular, was a great sin for Iranians [41]. In Persian poetry and prose, flowers are entwined as attractive, glamorous, and unique symbols of love and life, and reminders of heaven. Persian gardens are regarded as lush oases in the midst of vast deserts where water shortages and harsh winds are the enemies of plants.

In contrast to this apparent nature-loving affiliation of Iranians, unfortunately, less favourable or nil narrations exist to depict Iranian desires for respecting wildlife. Disturbing narratives in the historical records have revealed the dark and shocking mass killings of wild animals in the name of hunting for leisure. As early as the Sassanid era (ca. 600 A.D.), evidence has been found of hunts for Persian fallow deer and wild boar at Taq-e-Bustan (West Iran) [46]. The handful of photos that remain from those Iranian kings who lived about 150 years ago (Qajar dynasty) portray a grumpy and arrogant king holding a gun in his hand, and standing proudly on the top of hundreds of massacred deer. A translated narration from that time illustrates one report: “Six thousand pheasants, 150 deer, 63 buffaloes, 35 tigers and 18 leopards were the results of one day’s hunting in Miankaleh [now a protected area in Mazandaran]. Masood Mirza (Zellolsoltan), a son of Nasereldin-Shah [king], hunted down all these with a group of his friends” ([41], p. 49). However, not on such a scale, reckless hunting of mammals, carnivores, and birds for leisure has always been a part of the lifestyle of oligarchs, wealthy clans, and some rural residents in parts of Iran. The last Iranian endemic lion (*Felis leo persicus*) and tiger (*Panthera tigris tigrus*) species were observed in the wild in 1942 and 1958, respectively [46]. Once endangered species become extinct through lack of public awareness or concern, efforts to reverse wildlife losses may be difficult or impossible.

### 1.3. Newspapers in Iran

In Iran, the history of printed newspapers reveals that newspapers have grown alongside photography, which was started by the Frenchman Jules Richard, who arrived in Tehran in 1844 [47]. Mirza Mohammad Saleh Shirazi, Iran’s first newspaperman, launched the country’s first newspaper called Kaghaz-e Akhbar (literally ‘The Newspaper’) in 1837 [48]. The paper was inaugurated when the literacy rate in Iran was around 5% [49]. In comparison, other communication tools were not introduced until a century later: the first wireless telegraph service and radio broadcasting were established in Tehran in 1930 and 1940, respectively [48].

Newspapers are still a dominant media in developing countries as conduits for informing people about daily news. This is perhaps due to inadequate access to the internet or lack of digital skills of the older generations within these countries (e.g., [50]). Our research is based on a selection of the Iranian print newspapers, which are most favoured by the public. Building on the long history of newspaper publications in Iran, most newspapers are published in the Persian language on a daily basis. Additionally, there are a few newspapers published in English. Newspapers are normally produced and distributed nationally, although some are published to cover local news related to a specific city or province. Circulation rates of print-based newspapers vary in Iran, though recent trends show a general decline due to the rise of online and digital media or social networks [51]. Nearly all newspapers release an online version as well. Apart from the Persian content, newspapers use material translated from other languages (mostly English) from global news outlets.

In addition to thematical newspapers and journals published to cover specific subjects, such as the economy, regular newspapers typically contain political, social, sports, economic, and environmental news items, as well as promotional material and advertisements. Some news items are illustrated with colourful pictures, and others with tables or charts. Iranian daily newspapers allocate parts of their pages to environmental news on a diverse range of matters related to water, forests, rangelands, and wildlife.

This research focusing on wildlife was part of a broader project to evaluate environmental news items in three major newspapers, namely Hamshahri, IRAN, and Jam-e Jam [52]. The print versions of these three newspapers published in Persian were examined for seven years from 2007 to 2014. Two of these newspapers (IRAN and Hamshahri) are dominant players in Iran’s daily press market [53]. Hamshahri is published in Tehran (Capital city), although it also contains national and international news. The other two newspapers embrace a more comprehensive geographical scale and focus on the national level. Throughout this research, the Persian names of newspapers are used, except for one newspaper in which a word with uppercase letters was used (IRAN), to distinguish it from the country’s name (Iran).

## 2. Data and Methods

### 2.1. Data Collection

Dataset: the intention of the research was to identify as many news items as possible from the selected print newspapers, when, at least a small proportion of their pages (10% of a full page) were allocated to environmental topics. All environmental news items were read carefully (at least twice). A total of 434 articles were found relevant to environmental news, with topics such as water, forests, and wildlife. From this pool of 434 news items, those with ‘wildlife’ as a subject were screened and assigned a code. Accordingly, 61 news items were found to cover the specific subject of ‘wildlife’.

Database: a spreadsheet table composed of several columns (‘fields’) was developed for the research. We defined nine fields, and for each coded news item (‘record’), corresponding information was entered separately into the spreadsheet table (Table 1). At the end of the data entry process, a total of 915 entries composed of 61 news items (records) × 15 indicators (fields), having wildlife topics, were extracted from news items, cross-checked, and entered into a spreadsheet to form our database (Table 1).

### 2.2. Data Analysis

A database is an organised collection of data that are stored in tables, as the main structures of the databases [54]. A relational database management system (RDBMS) utilises the commands of Structured Query Language (SQL). Based on data stored in rows (records) and columns (fields), users can obtain results from queries directed to one or multiple tables at the same time. A series of rules, constraints, and filters are applied to modify queries, while data can be retrieved and updated in a RDBMS at any time [55]. These sophisticated capabilities make the computation and management of datasets easier to handle by natural scientists as they generate massive datasets during large-scale natural resource inventories [55]. The application of SQL in wildlife research is rare: we found only one example, in which SQL was employed directly in assessing the illegal trade of pangolins and arapaimas in the form of leather products imported to the USA [3]. Globally, a variety of measures are used in media analyses, such as article coding [56], framing (e.g., [57]), or content analysis (e.g., [58]). These existing approaches cannot establish links between unrelated fields [59]. In our research, the prepared database was exported into a SQL-based database management system, and various queries were made based on the rules of a relational database management system. In some cases, we made simultaneous four-field relationships (message/contributor/spatiality/allocated space) in our SQL queries. Without SQL coding in place, extracting such multiple associations from 915 cells would not be feasible, revealing the advantages of our approach.

## 3. Results

A summary of query results is presented in Table 2.

### 3.1. Proportion of Wildlife News in Newspapers

The Hamshahri newspaper was the disseminator of wildlife news with the greatest coverage. Despite its geographical circulation being limited to Tehran city (Capital), Hamshahri had substantially higher numbers of wildlife news items (67% of the total) than the other two newspapers. As a local newspaper, Hamshahri could view its primary obligation to distribute more local news than translated international news. Personal field observations and communications (FA) confirm that the Hamshahri newspaper is still the first option for those interested in reading environmental news in general.

### 3.2. Wildlife Topics

Topics covered national to international themes, as illustrated in Table 3. The most significant numbers of news items related to endangered land species and global conservation. Endangered native species in Iran that were cited in news items included the Asiatic cheetah (*Acinonyx jubatus venaticus*), Persian onager (*Equus hemionus onager*) and Persian fallow deer (*Dama mesopotamica*). Asiatic lions (*Felis leo persicus*) and Caspian tiger (*Panthera tigris tigris*) were listed as extinct species, although Asiatic lions were reintroduced to Iran in 2019. In total, articles dealing with land and marine endangered species represented 35% of wildlife items, of which the Hamshahri newspaper reported 84% and IRAN the remainder. One of the sampled newspapers, Jam-e Jam, thus, did not include any items on national or international endangered species over the 7-year study period.

### 3.3. Messages

We considered three overall messages, as being ‘educating, alarming, or public awareness’ in assessing wildlife news (Table 2). We found that most published articles focused on public awareness (51%), followed by the alarming (39%) category.

### 3.4. Contributors

Most news items were authored by columnists (46%), followed by authorities (39%) (Table 2). Researchers had the lowest contribution (15%). ‘Authority’ was defined as a person who held a high-ranking decision-making post in an organisation. Surprisingly, researchers did not show much interest in writing articles for wildlife news in these newspapers.

### 3.5. Spatiality

In this research, most items targeted wildlife news at the local scale (43%), followed by the international (26%) level (Table 2). Local wildlife news was published primarily by the Hamshahri newspaper. International wildlife news had short publication longevity (2010–2013 only) compared to other news items. To some extent, the animal species (e.g., elephants) covered by international news were irrelevant to the dominant species contributing to Iran’s wildlife biodiversity, and that may have limited public interest.

### 3.6. Allocated Space

We viewed ‘allocated space’ as referring to the proportion of space allocated to wildlife news on one page, which varied from 0.1 (10%) to 1 (100%). The results showed that most news coverage was limited to a half-page or less (10% space for half of the cases) (Table 2). In only one instance was a full page allocated for a single news item and this was authored by columnists in the Hamshahri newspaper. The sole full page news item was related to the extinction of three animal species at the national level, while one of the half-page news items was related to an urban bird garden at the local level.

## 4. Discussion

### 4.1. Analysing Wildlife News in Iranian Newspapers

Here, we will return to the questions that were outlined in the ‘Introduction’ section.

#### 4.1.1. Are Local Experts Involved in Writing Technical Articles or Are These Articles Just Being Translated from International Sources?

Generally, experts were not involved in writing technical articles: columnists and authorities had the highest contributions. We also found that some news items were translated from English language websites. Comparatively, the contributions of authorities in this research (39%) was in agreement with that reported for Estonian newspapers (40% for a study period of 1992–93) [60]. There is a tendency among journalists to rely on ‘most easily sourced’ information, such as that provided by government agencies, in order to respond rapidly to their media organizations [23]. The extent of engagement and availability of personnel employed at universities and research institutes would also influence journalist behaviours in news sourcing.

Researchers (with 14% share) were not the main contributors in writing wildlife articles for the sampled newspapers. Similarly, it was found that researchers had the lowest contributions in writing general ‘environmental’ news items (10%) [52] and ‘water’ news items (9%) in Iranian newspapers [59]. These findings are in accordance with those reported by Rust [61], who found that academics did not show interest in writing news on the human–wildlife conflict in Namibia (2% share). In Brazil, another developing country, scientists’ contributions to writing news items was low, from 6.2 to 8.2% [62].

Overall, this low contribution of researchers in Iranian newspapers is not solely limited to wildlife articles. When assessing newspaper items relating to the relocation of Tehran as a capital, it was found that only 19% of contributors were ‘academics’ [63]. Surprisingly, though, Lemańczyk [53] found that 73% of news contributors who reported on nanotechnology in the Hamshahri newspaper were ‘scientists’. Given that columnists may regard nanotechnology as a highly technical and specialised scientific field in which they did not have expertise or existing contacts, they may have sought authorities and researchers for input. Iranian researchers and university lecturers generally do not consider newspapers to be either potential sources of scientific information or outlets for dissemination of their research findings. This minimal involvement relates to the lack of recognition of newspaper articles as an element, adding value for academic staff promotion in Iranian universities, although granting bodies in other countries are increasingly requiring evidence of raising community awareness as a research outcome. Nevertheless, some columnists may use interviews or scientific publications (from researchers) as primary sources of their contributions to newspapers.

The issue of a contributor can also be viewed from another perspective—that of trust: in particular, does the public trust information from non-researchers who write on environmental subjects? In transmitting environmental information, it has been emphasised that the nature and foundations of public trust in different actors, the so-called credibility gap, must be understood [64]. Trust in local officials is absent in many countries and depends on the success of officials in delivering public goods, such as employment [65]. However, trust between citizens and public officials has proven to be effective during the evacuation of people in the case of natural disasters [65]. In contrast to the low direct contribution of scientists to news items in Iranian newspapers, Olausson and Berglez [66] showed that the media had a high dependency on scientific experts, and technical knowledge was still an essential aspect of news coverage (e.g., see [18], regarding endangered panthers in Florida). Effective and meaningful communications with the public and establishing a partnership between scientists and newspapers are recommended in biodiversity conservation [28]. Although not currently available in an Iranian edition, The Conversation (https://www.theconversation.com (accessed on 18 April 2021)) is an online forum that would be a valuable innovation if adopted in the country, as it would encourage timely, ‘expert’, and publicly available scientific information, while also providing a useful resource for newspaper columnists.

#### 4.1.2. What Was the Message of an Article in Disseminating Wildlife News?

This research categorised the messages into three classes—public awareness, alarming, and educating.

Awareness: generally, awareness is affected by external factors, but it does not necessarily lead to behavioural change [10]. Here, the results showed that the majority of articles focused on public awareness (52%). However, a local Iranian survey, conducted on the level of knowledge of Shiraz residents on the Bamou Reserve Park (30 km from the city), found that the role of local media (newspapers, TV channels) in public awareness was insignificant [67].

Alarming: the ‘alarming’ category had a 39% share (although recording less than the ‘awareness’ message class). In the UK and Sweden, a similar result found that alarming news accounted for 34.9% based on a 2-month assessment of newspapers reporting all hazards [68]. In news items on near-threatened leopards in India, themes of conflict with humans dominated those of conservation (by three times) and educational (by four times), and included a headline of “Leopard population up, Shirur in grip of fear” [69]. Although such alarming and negative headlines are unlikely to induce calm, they may generate awareness and caution. However, it is said that inducing fear (by alarming people) can change attitudes, but may not alter behaviour [10].

Educating: the lowest numbers of wildlife items in our sampled newspapers were related to the message described as educating. In earlier research on ‘general environmental’ and ‘water’ news items, it was noted that the educating news items were the lowest, with 9% [52] and 2% [59], respectively. Media outlets must play a role as educators as part of their essential functions [19], though they must be treated with caution when considered for public education about wildlife [23]. Delclaux and Fleury [70] stressed the need for newspapers to aid with communication between special interest groups and, thereby, promote public dialogue and enhance understanding.

#### 4.1.3. How Much Space Is Allocated for Wildlife News in a Typical Iranian Newspaper?

Here, we showed that spaces allocated for the majority of news items were less than half a page. None of the news items was presented on the first page of any newspaper. If we assume that a correlation exists between the importance and attraction of news with the space it is allocated, then it is safe to say that wildlife news in Iranian eyes is almost insignificant. Its contribution can be compared negatively with sports news published in a regular Iranian newspaper, where more than a page is devoted to a single football game [71].

An essential part of any business is income generation and, for the newspaper industries, income and profit originate from advertisements or selling news to readers/customers, as indicated for the UK [72]. As a result, they are unwilling to allocate space for news items that are not deemed essential or tradeable. However, the three newspapers that were analysed here receive full or partial financial assistance, or subsidies from public funds; therefore, gaps produced by a lack of wildlife news reports are not intended to be filled by advertisements.

#### 4.1.4. What Is the Spatial Focus of Wildlife News in the Newspapers?

The majority of wildlife news items were related to incidents that occurred at the local level (43%). For instance, a modest item of news (20% of a full page) in IRAN referred to carp fish deaths in a small spring located in a small village in central Iran. Such news is unlikely to attract national audiences, but, surprisingly, it was published in IRAN, a newspaper with a national circulation. Whether this item filled a gap due to a shortage of other news is not known. Other research conducted on the role of newspapers in transmitting environmental news in Iran also found that the highest proportion of news items were related to the local level (46%) [52]. In another instance, however, local newspapers were not functioning to the satisfaction of their public. Based on a survey of local knowledge of the Bamou Reserve Park in Shiraz (Iran), one issue raised by respondents was the inappropriate coverage of this park in their local newspaper, which insisted on showcasing it to the public through excessive promotion [67]. Focusing on (local) regional and suburban distribution news was also evident in relation to items on a native bird in Australia (the Magpie, which swoops on people during the nesting season), with inclusion mainly restricted to the regional and suburban press [23].

On a broader scale, our research showed that wildlife news published at the national level constituted 23% of news items and that 26% of wildlife news items were related to the international category (Table 2). As a comparison, the figures for general environmental news published (1992–93) on local issues in Estonia and Latvia were 74% and 86%, respectively [60]. In other news items in Iranian newspapers, generally, the international category was also important. Coverage of international sports events constituted 30% of items in sports newspapers over a 3-month period [71], and 63% of news coverage on research in nanotechnology reported foreign research findings [53].

The presence of international news in local and national newspapers, and their relevance to national and local incidents, is debatable, as usually nearby events receive the most coverage [34]. Hungerford and Lemert noted, perhaps for the first time, the relevance of distance to news items, citing “a presumed greater severity of environmental problems afflicting regions outside of a newspaper’s home region” ([33], p. 4). This suggests that people, on occasion, may prefer to interpret problems in other places as being worse than their own, thus providing a rationale for their own inaction.

### 4.2. Wildlife Conservation Efforts and Interest in Iran

The narratives of Iranian attitudes to nature, which were outlined earlier, do not depict an improved situation for wildlife in contemporary Iran. Hunting for leisure has been a severe threat to the wildlife population in mountainous rural areas where hunting has even turned into an alternative livelihood for the destitute, unemployed segment of the population. A diverse range of wild animal species are sold illegally in local and international markets. New overseas markets are appearing, with some residents of the Persian Gulf countries willing to pay substantial money in situations where keeping exotic species is becoming a celebrity trend [31]. One of the most active online Iranian sales websites reported a staggering 1,208,000 advertisements for animal trades in just two months. The most significant shares of these advertisements were related to birds (50%), farm animals (27%), dogs (9%), and aquarium fish (6%) [73]. The Iran Department of Environment reported the release of 340 birds, 279 mammals, and 1920 fish species illegally obtained and confiscated in just one province for one year (2021). Their staff also seized over 11,000 hunting tools, such as illegal hunting guns, traps, cages, knives, and hunting paraphernalia for the same year [74].

Wildlife conflict studies in Iran are rare, and so are the solutions. One domestic survey conducted across three central provinces showed that the human–onager conflict relates to the depredation of crops, such as alfalfa, wheat, and barley [75]. Another Iranian study used the information from GPS-collared wolves to evaluate the feeding behaviour of wolves and the locality of human settlements in Hamedan province (West) [76]. A 15-year study using the mortality data of Persian leopard showed that more than 60% of unnatural mortality was due to targeted killing by poison bait, shooting by ranchers, trophy hunters, and military forces, followed by road collision (26%) [77]. In another Iranian wildlife survey, collision with vehicles was recorded as the primary reason for carnivores’ mortality (including cheetah) during 2007–2015 [78].

Undoubtedly, journalists like to highlight and sometimes exaggerate situations by providing controversial or dramatic headlines or events that capture the attention of their readers [79]. Modern journalists who live in the digital era use more sophisticated techniques and digital archives to communicate information and facilitate maximum comprehension, including “presenting or arranging graphical elements in a perceptually advantageous manner” ([80], p. 304). However, the shocking images of starving bears, lions, and monkeys in the zoos disseminated by personal blogs and the stories of social networks reveal how precarious the living conditions of animals are in certain zoos in Iran. For instance, a report from an online Iranian news agency known as ‘ISNA’ portrays a weakened lion staring at a camera from a small cage in a zoo located in one of the largest cities in Iran [81]. This photo highlights the possibility of other animals’ terrible living conditions, likely kept in such cages with limited spaces, or being inadequately fed. In Iran, as elsewhere, “the media plays a powerful role that will either further [wildlife] conservation or leave it as a neglected element of our heritage” ([82], p. 52).

Currently, efforts have been boosted to conserve wildlife species in Iran despite climate change challenges [83]. For instance, cheetah conservation is a unique and successful story in Iran’s animal conservation arena. Like other big cats in the country, the cheetah is threatened by anthropogenic impacts, including habitat degradation and a declining number and diversity of prey, and by droughts in recent decades [84]. The habitats of this valuable and rare species are predominantly located in the central deserts. Accordingly, a collaboration was instigated in 2001 between Iran Department of Environment and UNDP to save the endangered cheetah in Iran [85]. The result of such enhanced conservation activities demonstrated an increase of 17% in the prey population combined with a decrease in poaching violations by 27% in cheetah habitats in 2013–2014 [85]. In order to discourage the killing of cheetahs, the Department of Environment implemented a monetary compensation scheme of direct payments to herders whose livestock are attacked and killed by wildlife, though compensation is restricted to a few special cases [75].

Animal–human interactions occur in the wild, in conservation areas, in zoos, and in pet-keeping. Having captive animals as guardians, companions, or status symbols has blossomed in recent years, and is not limited to Iran’s urban areas, although the new middle-class generation now sees pets (primarily dogs) as part of their lifestyles, despite discouragement from older generations (parents) and public attitudes. In other Middle Eastern countries, younger generations and celebrities keep, in captivity, a diverse range of exotic species (e.g., tigers, gorillas, dolphins, etc.) [31]. This largely unregulated behaviour unlikely benefits wildlife conservation in natural habitats.

## 5. Conclusions

Analyses of news items are rare in Iran, so our research on Iranian newspaper reporting can augment the existing paucity of coverage in this field. Both the quality (use of translated general material, relevance to Iran of the species reported, low contribution of researchers) and quantity of wildlife news in selected Iranian newspapers are inadequate. Spanning a 7-year period of mainstream newspaper coverage, this research concluded that the presentation of overall wildlife news in the country’s newspapers is deficient, with an annual average of eight news items for three newspapers combined. The findings also showed the low contribution of researchers to wildlife news. This aspect needs more scrutiny from scholars in the communication and journalism fields. Although the timeframe in this study (2007–2014) coincided with essential and challenging news regarding the nuclear issue in Iran, what is clear is the low level of attention given by Iranian newspapers to wildlife issues.

Iranian newspapers must disseminate a greater number of wildlife news items to attract general public awareness about wildlife in the country. Relevant global literature suggests that people’s overall perceptions on the management and importance of wildlife are diverse and uneven, varying in accordance with lifestyle, education, culture, awareness, pet ownership, and previous personal experiences. For Iran, however, sociologists, anthropologists, and psychologists must respond to this social complexity if wildlife conservation is to succeed. Unfortunately, to the best of our knowledge, such analytical attempts to scrutinise the behavioural relationship between individuals and animals in Iran are rare if they exist at all.

Global research on wildlife coverage in the media, whether online or in newsprint, remains insignificant, and our study for Iran displayed limited topics, methodology, and species (mostly carnivores in Africa). Newspapers are an under-utilised resource for engaging the public in wildlife conservation. Wildlife needs to become part of media analysis and research at various scales, in different countries and social contexts, and encompassing diverse media platforms. This will assist in ensuring current and comprehensive public perceptions of wildlife and shape the development of relevant policies and guidelines for wildlife conservation.

## Figures and Tables

**Table 1:** Structure of the database developed in this research.

Field	Value	Definition
Title	IR	‘IRAN’ newspaper
	HA	‘Hamshahri’ newspaper
	JA	‘Jam-e Jam’ newspaper
Date		Date of any news items
Subject	Wildlife	Any news items related only to wildlife (e.g., birds) from other environmental news
Description		A few descriptive words in Persian extracted from the title of news item (e.g., extinction of cheetah)
Message	PA	‘Public Awareness’
	ED	‘Educating’
	AL	‘Alarming’
Contributor	C	‘Columnist’
	R	‘Researcher’
	A	‘Authority’
Spatiality	L	‘Local’
	P	‘Provincial’
	N	‘National’
	I	‘International’
Allocated space	0.10 to 1	The proportion of space allocated to wildlife news on one page varying from 0.1 (10%) to 1 (100%)

Data types are based on standard data categories defined by SQL. ‘Null’ as a value was not a case in our research. ‘Authority’ was defined as a person who held a high-ranking decision-making post in an organisation.

**Table 2:** Summary of queries in this research (the numbers are the number of news items per sampled newspaper).

Newspaper	Message			Contributor			Spatiality				Allocated Space		
	Public awareness	Alarming	Educating	Columnist	Authority	Researcher	Local	Provincial	National	International	Half-page or more	10–40%	<10%
Hamshahri	19	17	5	21	11	9	17	0	9	15	5	31	5
IRAN	11	6	1	5	13	0	10	4	3	1	0	17	1
Jam-e Jam	1	1	0	2	0	0	0	1	1	0	0	2	0
Total	31	24	6	28	24	9	27	5	13	16	5	50	6

**Table 3:** Categories of wildlife news items.

Category	Topic	%	Category	Topic	%
Land	Endangered species	30	Marine	Endangered species	5
	Extinction	8		Invasive fish species	2
			General	Migratory birds	5
	Birds	5		Global conservation	24
Others		5		National law	5

Notes: ‘Others’ include other local wildlife news not classifiable as land, marine or general. ‘General’ wildlife items did not specify whether migratory birds were land or marine species.

## Data Availability

The data presented in this study are available upon request from the corresponding author.
